# No effect of moderate alcohol intake on the detection of concealed identity information in the laboratory

**DOI:** 10.1038/s41598-020-76811-x

**Published:** 2020-11-19

**Authors:** Kristina Suchotzki, Heidi May, Matthias Gamer

**Affiliations:** 1 Social and Legal Psychology, Institute of Psychology, University of Mainz, Binger Str. 14-16, 55122 Mainz, Germany; 2 Department of Psychology, University of Würzburg, Marcusstr. 9-11, 97070 Würzburg, Germany

**Keywords:** Psychology, Human behaviour

## Abstract

The Concealed Information Test (CIT) enables the detection of certain (e.g., crime-relevant or personal) information, even if participants aim to conceal their knowledge. The current preregistered study investigated whether previously observed impairing effects of alcohol intoxication on participants’ performance in a reaction time CIT (RT CIT) field study also translate to a laboratory environment. In contrast to the previous study of Suchotzki and Gamer (Sci Rep 8:7825, 2018) in which alcohol consumption was voluntary and self-administered, the current study used a randomized assignment of participants to either an alcohol group (*n* = 88; receiving a drink with 3 cl alcohol) or a sober control group (*n* = 89; receiving a drink with just some alcohol drops to hide group assignment). After drink administration, participants completed an RT CIT, in which they were instructed to hide knowledge of their own identity. Blood alcohol concentration (BAC) was estimated via breath alcohol ratio. In contrast to the previous field study, results revealed no differences in CIT-performance between intoxicated and sober participants. Aside from questioning the robustness of the result of the previous field study, our results also point to a number of interesting theoretical explanations for the discrepancy between both results, which are elaborated in the discussion.

## Introduction

Imagine you went to a party and talked just a little too long to someone you found attractive. You also had a drink or two. Then you return home and find your partner still awake, asking you: “How was it, did you meet anyone interesting?”. What do you think? Do the drinks make a slip of your tongue more or less likely? Or, to phrase it in scientific terms: Does alcohol intoxication have an effect on the processes of deception and information concealment?

Empirical data on this question are scarce and mixed. A frequently used test to assess information concealment is the Concealed Information Test (CIT^1^). In this test, the examiner asks a question (e.g., “What was stolen?” in case of a theft investigation) and presents the examinee with the critical answer (e.g., “a painting”) and several neutral response alternatives (e.g., “a necklace, money, a watch, a notebook”). Using physiological or behavioral indices, one assesses altered responses to the previously encountered (yet concealed) critical information compared to the other neutral details. Interestingly, Bradley and Ainsworth^2^ found that alcohol intoxication during a mock crime reduced the effectiveness of a CIT, most likely driven by poorer encoding of the relevant information (but see^3^, who could not replicate this effect). In a more genuine deception paradigm, Kireev et al.^4^ observed diminished behavioral (i.e., reaction times) and neural (i.e., event-related potentials) differences between lying and truth telling in intoxicated compared to sober participants. However, these results should be evaluated with caution due to the very small sample size of *n* = 13. Similar results were not observed in a larger field study by Suchotzki et al.^5^, in which self-administered alcohol intoxication (during a science festival) did not correlate with the speed of deceptive (vs. truthful) responses. In a similar field setting in which participants freely choose whether and how much alcohol to consume (a bar and night club), Suchotzki and Gamer^6^ did, however, observe a correlation between the amount of alcohol consumption and effects in a reaction time CIT (RT CIT). In this study, a role-play cover story was used and participants were instructed to try to prove their potential as “the new James/Jane Bond” by taking on a new identity and denying recognition of their own personal information (name, birthday, birthplace, names of the parents). This type of personal information was used to ensure that even intoxicated participants would still remember it and thereby diminish the influence of hampering effects of alcohol on memory. Results were in line with the idea that acute alcohol intoxication impairs cognitive functioning, as higher alcohol intoxication correlated with enlarged RT CIT-effects (i.e., differences between deceptively denying knowledge of their own personal information compared to truthfully denying knowledge of irrelevant information). They also fit with the popular idea that increasing cognitive load interferes with deception and information concealment (e.g.^7,8^). But despite the advantage of higher ecological validity, such field studies come at the cost of a diminished control of potentially confounding factors. Specifically, correlations between habitual alcohol consumption and acute alcohol consumption indicated that participants who drink more on such occasions are also the ones who drink more in general^5^. This is relevant, as impairing effects of alcohol consumption on executive functioning, which may be responsible for enlarged effects in the RT CIT (for reviews see^9,10^), have not only been observed for acute but also for habitual alcohol consumption. The aim of the current study was therefore to investigate whether the effects of acute alcohol intoxication on the RT CIT-effect observed in Suchotzki and Gamer^6^ can be replicated in a controlled laboratory context with a randomized assignment of participants to an alcohol or a sober control condition.

## Methods

Hypotheses, sample size, design specifications and analysis steps were preregistered before data collection on https://osf.io/jced3.

### Participants

Power analyses (conducted with G*Power) revealed that a sample of 79 participants was required in order to achieve a power of 0.95 to discover a correlation between alcohol level and the RT difference between probe and irrelevant items of *r* = 0.39 (i.e., the one found in Suchotzki and Gamer^6^). Due to its organizational effort (because of the alcohol administration), the current experiment was conducted together with another study. This other study required 182 participants to be tested, resulting in a power of 0.9998 to discover an effect of *r* = 0.39, while at the same time having the advantage to discover potentially smaller effects. Therefore, we preregistered a targeted final sample size of 182 participants (± 10%, taking into account potential variations in participant sign-ups and no-shows).

In total, 248 participants were recruited. Of those, 138 were recruited via the online participant pool of the University of Würzburg. Although this platform is generally accessible by anyone, the majority of participants in this system are students of whom most study psychology. The other participants were recruited via Facebook (through groups and networks of the two student experimenters), a mailing list for participants of the psychology department of the University of Würzburg and a local advertisement platform (https://www.wuewowas.de, frequented by the general public). Thus, the sample was not restricted to students, yet it can be assumed that the majority were students and among them mainly psychology students.

The study conformed to the principles expressed in the Declaration of Helsinki and was approved by the Ethics Committee of the Institute of Psychology of the University of Würzburg. Participation was voluntary and all participants provided written informed consent. Of the initial 248 participants, 62 did not fulfil our strict pre-registered inclusion criteria (due to the alcohol administration; see below) and were compensated and sent home after completing the initial questionnaires. 186 participants took part in the main experiment, receiving either 12 Euro or course credit for their participation. For our experiment, data of three participants were excluded because of an experimenter error during the experimental procedure. Data of six participants were excluded because of a high error rate in the CIT (exceeding the group mean error rate per item plus 2.5 *SDs*). The mean age of the remaining 177 participants was 24.21 years (*SD* = 5.33 years; 112 female, 65 male). Of those, 88 participants were assigned to the alcohol group, 89 to the control group. Age and gender for both groups can be found in Table 1. Group assignment was randomized, with the restriction that participants who were tested at the same time were always in the same group (to facilitate drink preparation).

**Table 1:** Means and Standard deviations of different variables for the alcohol and the control group and results of the Welch’s *t* tests.

Measure	Alcohol		Control		*t*	*df*	*p*	*d*
	*M*	*SD*	*M*	*SD*				
BAC (%)	0.04	0.01	0.00	0.00	26.75^a^	87^a^	< 0.001^a^	2.85^a^
Gender (% female)	62.50	–	64.04	–	0.05^b^	1^b^	0.83^b^	–
Age (years)	24.66	5.17	23.78	5.48	1.10	174.6	0.27	0.17
Tension	2.11	0.95	1.90	0.92	1.53	173.8	0.13	0.23
Boredom	3.15	0.69	3.18	0.70	0.33	173.9	0.75	0.05
Fatigue	2.99	0.94	3.33	1.00	2.33	173.3	0.02	0.35
Intoxication	3.05	0.86	1.52	0.74	12.70	170.8	< 0.001	1.91
BIS-11	59.31	7.42	60.39	7.74	0.95	174.9	0.35	0.14
AUDIT	5.63	1.68	5.52	1.79	0.39	173.2	0.70	0.06
DAST-10	0.24	0.46	0.33	0.56	1.14	168.7	0.26	0.17
PPI-R	366.67	22.75	365.49	21.13	0.36	173.7	0.72	0.05
Lies last 24 h	2.55	4.51	2.44	4.72	0.16	174.4	0.88	0.02
Lies last week	7.40	9.52	7.31	11.10	0.06	170.4	0.95	0.01
Subj. Alc. Group (%)	94.32	–	43.82	–	52.68^b^	1^b^	< 0.001^b^	
Test difficulty	2.21	0.87	2.38	1.01	1.25	172.1	0.21	0.19
RT CIT-effect (ms)	78.17	57.32	69.44	52.42	1.06	173.3	0.29	0.16
ER CIT-effect (%)	2.34	5.94	2.16	4.09	0.23	154.3	0.82	0.04

*BAC* blood alcohol concentration, *Subj. Alc. Group* percentage of participants who believed they were in the alcohol group, *RT* response time, *ER* error rate, *p* values reported two-tailed, *d* independent Cohen’s *d*.

^a^A one-sided *t* test was used to test the BAC in the alcohol group against zero.

^b^For categorical variables, Pearson’s chi squared (χ^2^) test was used.

### Procedure

Already during recruitment, potential participants were informed about a number of exclusion criteria like a regular intake of medication, the possibility of a pregnancy or an alcohol dependency. Furthermore, they were briefed that they would either receive an alcoholic mixed drink or a non-alcoholic one during the experiment. They were asked to choose their appointment in a way that at least 2 h elapsed after their last meal and they should take into account that they may not be allowed to drive depending on the BAC after the testing.

Testing took place at a lab, in which up to four participants could be tested simultaneously. The whole experiment lasted approximately 75 min and was usually scheduled between 11.00 and 18.15 h. After signing the informed consent, participants received a questionnaire assessing their health as well as their habitual alcohol and drug consumption using the Alcohol Use Disorders Identification Test (AUDIT^11^) and the Drug Abuse Screening Test (DAST-10^12^). Female participants additionally received a questionnaire assessing the possibility of a pregnancy. The experimenters immediately evaluated the questionnaires to determine inclusion or exclusion of the respective participants. Participants were excluded from the study (and thus sent home before the potential alcohol administration), if they indicated experiences of strong side-effects after consuming a moderate amount of alcohol or generally consume either less than two alcoholic drinks a month or more than eight alcoholic drinks a week. Values higher than 6 in the items b-j of the AUDIT or 2 in the DAST-10 also led to study exclusion. Further exclusion criteria were regular intake of medication, any acute or chronic physical or mental illnesses, and alcohol dependencies of either the participants themselves, or their family members. Female participants who indicated that they could not rule out a pregnancy were also sent home. We deliberately chose very strict exclusion criteria in order to exclude any risks of alcohol intake for the subjects. Participants who were excluded at this stage of the experiment (*n* = 62) received a compensation of 4 Euro or the respective amount of course credit. Participants with high values in the AUDIT (> 10) or DAST-10 (> 5) were handed written information about counselling and therapy options.

After questionnaire evaluation, the experimenter prepared the drinks for the respective group of participants. Participants assigned to the alcohol group received a drink containing 3 cl of high-percentage alcohol (96.6%) together with 250 ml tonic water and orange juice. Participants assigned to the control group received a drink containing 250 ml of tonic water, orange juice and non-alcoholic ginger beer. In order to mask group assignment, the experimenter also added half a teaspoon of the high-percentage alcohol on top of the non-alcoholic drink. Participants were asked to drink their beverages within the next 5 min. Start and end time of the drinking were noted by the experimenter. During this period, participants filled in questionnaires on the PC assessing their demographic variables as well as the time of their last larger meal. Subsequently, trait impulsivity was assessed with the Barratt Impulsiveness Scale (BIS-11^13^) and psychopathic personality traits with the Psychopathic Personality Inventory-Revised (PPI-R^14,15^). Furthermore, a questionnaire about participants’ self reported lying frequency during the last 24 h and during the last week was administered^16^. Afterwards, with a minimum of 25 and a maximum of 40 min after drink consumption (to allow the BAC to rise sufficiently, yet without being on the decline again), participants took part in the first part of the experiment consisting of six different moral dilemma scenarios. This paradigm (as well as a few additional questionnaires) was part of a separate study by a different research group and unrelated to the current one. Then, participants completed the CIT, which was very similar to the one used in Suchotzki and Gamer^6^ and is described in more detail below.

After completion of the CIT, feelings of tension, boredom, fatigue, intoxication and perceived test difficulty were assessed using 5-point Likert scales, while drug use on that day (except for alcohol, cigarettes and coffee) was reported using a yes/no answer format. Participants were also asked to indicate whether they believed to be in the alcohol or the control group. Testing took place on four PCs, thus a maximum number of four participants were simultaneously tested. At the end of the experiment, blood alcohol concentration (BAC) of each participant was estimated with the Dräger Alcotest 3000, which converts the breath alcohol ratio into BAC in ‰, and the value was communicated to participants. Participants received a written debriefing outlining the design and basic questions of the study, as well as the information to refrain from riding a bike or driving a car in the next hours in case participants belonged to the alcohol group. Participants were also offered the opportunity to stay on site in a room designed for that purpose and were encouraged to mention any feelings of nausea or uneasiness to the experimenter (none of the participants did so). Finally, participants were thanked for their participation and received their compensation.

### Reaction time concealed information test

The RT CIT including its introduction and a pre-test procedure were presented with Inquisit 4. The whole procedure closely resembled the RT CIT used in Suchotzki and Gamer^6^. It began by explaining to the participants that they had to prove their potential for being a good secret agent (the “new James/Jane Bond”) and therefore, they had to successfully take on a new “cover” identity and hide their true identity. Participants were first asked to truthfully indicate the five identity stimuli that they would have to hide in the following test (and that served as probe items). This was then followed by a pre-test procedure, during which participants were presented with five stimuli belonging to their new “cover” identity. These stimuli served as target stimuli during the test and were “Name: Silvia/Peter” (depending on the sex of the participant), “Birthday: 8. May”, “Place of Birth: Lübeck”, “Name of mother: Ulrike”, “Name of father: Erich”. Participants were told to remember these stimuli thoroughly, so that they could successfully enact this person during the following test. A screen with all five stimuli was presented twice for 30 s. Post presentation, participants had to type in all five stimuli via the keyboard. In the RT CIT, participants were then told that the experimenter was aiming to “uncover” their real identity. Participants were instructed that they would see a number of identity stimuli for which they had to indicate whether they recognized them or not. Recognition should only be acknowledged for the previously learned target stimuli (by pressing the “yes” key) and denied for all other identity stimuli, including the stimuli referring to their actual identity (by pressing the “no” key). The keys for “a” and “l” of a standard QWERTZ keyboard were used, with the assignment of “yes” and “no” responses being counterbalanced between participants.

In total, 30 different stimuli (the five aforementioned target stimuli, five probe stimuli consisting of the actual identity stimuli of the participants and 20 neutral stimuli [i.e., names, dates and German towns]) were each presented six times in completely randomized order (180 trials in total). Reminder labels for “yes” and “no” responses appeared on the left and right lower part of the screen. Participants were instructed to respond as fast and as accurately as possible. If participants did not respond within 2500 ms, the words ‘too slow’ were presented centrally on the screen. In accordance with Suchotzki and Gamer^6^, this relatively long response deadline was chosen to allow for a potential general slowdown in RTs due to alcohol intake. The inter trial interval was set to vary randomly between 500, 600, 700, 800, 900 and 1000 ms. After 90 trials, participants could take a self-paced break^6^.

## Results

Data were pre-processed with R software and raw data, the preprocessing script and the aggregated data can be retrieved from https://osf.io/g3t7q/. The aggregated data were further analyzed with JASP (https://jasp-stats.org). Trials exceeding the response deadline were excluded first (0.88%). For the analysis of the RTs, also error trials (4.18%) and RT outliers (2.77%; RTs > 2.5 SDs from the mean per subject and item type) were removed. For *t* tests, the standardized mean difference *d* was calculated as a measure of effect size. For paired *t* tests, *d* was corrected for the intercorrelation of values (Cohen’s *d* for paired data). Partial ƞ^2^ values are reported as effect size estimates for analyses of variance (ANOVAs). Means and standard deviations of the different assessed variables for both groups can be found in Table 1.

As can be seen in Table 1 and in accordance with our manipulation, the average BAC in the control group was 0.00% (*SD* = 0.00%; range = 0.00–0.00%). The average BAC in the alcohol group was 0.04% (*SD* = 0.01%; range = 0.01–0.07%) and significantly exceeded zero with *t*(87) = 26.75; *p* < 0.001, *d* = 2.85. Additionally confirming the effectiveness of our alcohol manipulation, participants in the alcohol group reported a significantly higher feeling of intoxication than participants in the control group, with Cohen’s *d* indicating a very large difference between both groups. As expected due to our random allocation of participants to groups, no significant differences between groups were observed in gender, age, BIS-11, DAST-10, PPI-R, AUDIT and the number of self-reported lies within the last 24 h and the last week. Interestingly, while there were no significant differences between reported subjective feelings of tension, boredom and test difficulty, participants in the control group reported a significantly stronger feeling of fatigue after the experiment. The subjective guesses of group affiliation showed that our blinding procedure (with adding a very small portion of alcohol also in the control group) worked to a certain extent: approximately 44% of participants in the control group believed to have been in the alcohol group, while 94% thought so in the alcohol group.

Mean RTs and mean error rates (ERs) in all four conditions can be found in Table 2. To investigate the link between BAC and the CIT-effect, 2 × 2 AVOVAs were computed with the between-subject factor group (alcohol vs. control) and the within-subject factor item (probe vs. neutral) for both RTs and ERs. The ANOVA on RTs revealed a statistically significant main effect of item, *F*(1, 175) = 319.68, *p* < 0.001, ƞ^2^ = 0.65, with slower RTs for probe compared to neutral items. There was no significant main effect of group, *F*(1, 175) = 0.31, *p* = 0.58, ƞ^2^ = 0.00, and no significant interaction effect of both factors, *F*(1, 175) = 1.12, *p* = 0.29, ƞ^2^ = 0.00. The ANOVA on the ER revealed a similar pattern with a statistically significant main effect of item, *F*(1, 175) = 34.52, *p* < 0.001, ƞ^2^ = 0.17, with a higher ER for probe compared to neutral items but no significant main effect of group, *F*(1, 175) = 0.61, *p* = 0.44, ƞ^2^ = 0.00, and no significant interaction effect of both factors, *F*(1, 175) = 0.05, *p* = 0.82, ƞ^2^ = 0.00.

**Table 2:** Mean reaction times and error rates in all four experimental conditions.

	Reaction time (in ms)		Error rate (in %)	
	Probes	Neutral items	Probes	Neutral items
Alcohol group	639.29 (82.75)	561.13 (65.11)	3.06 (5.77)	0.72 (1.49)
Control group	628.92 (82.02)	559.48 (75.99)	2.67 (3.88)	0.51 (1.26)

Standard deviations are given in brackets.

**Figure 1:**
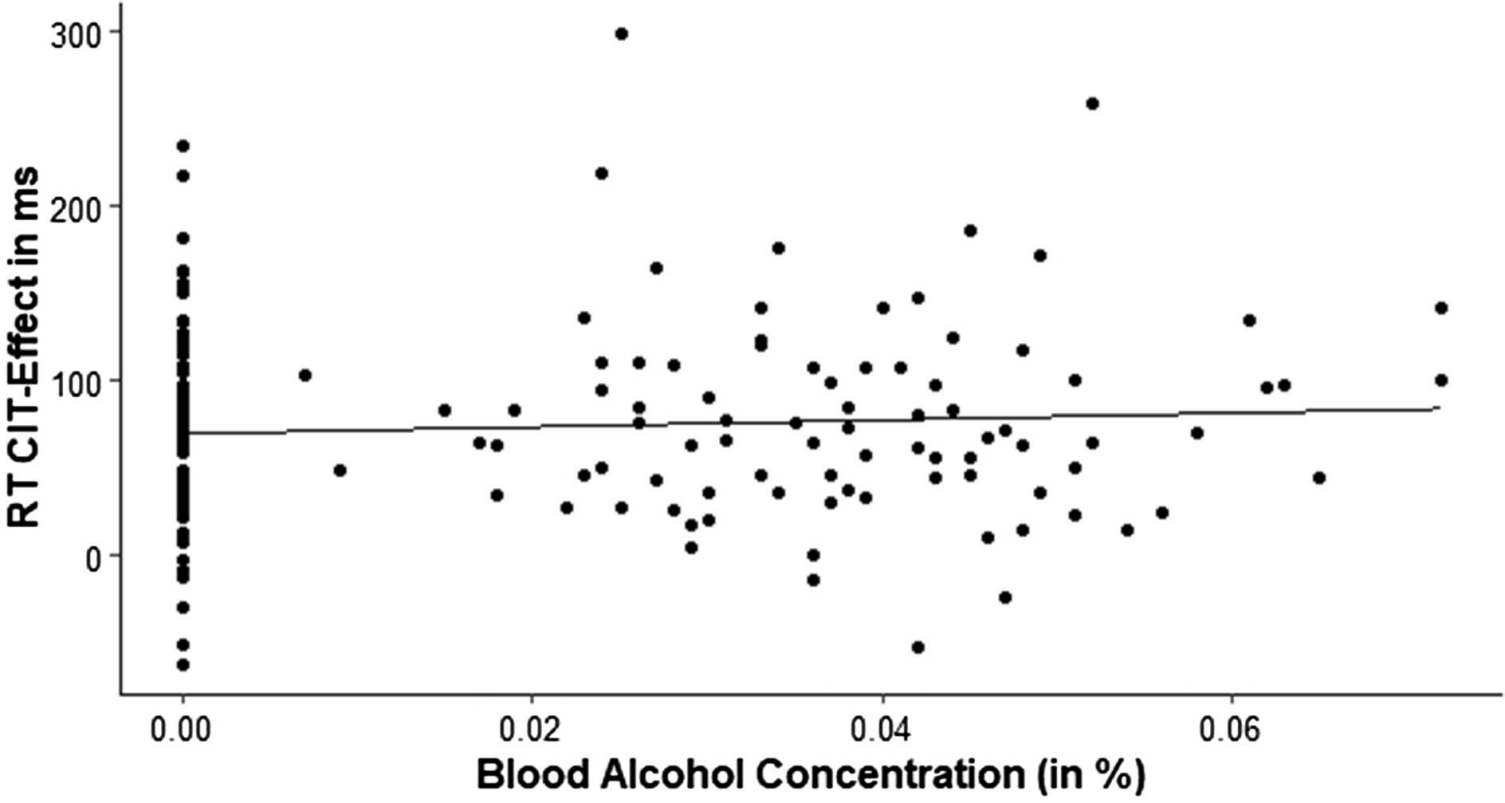
Scatter plot displaying the mean blood alcohol concentration in relation to the mean reaction time CIT-effects.

For a better comparison with the data of Suchotzki and Gamer^6^, we additionally computed the correlations between BAC and CIT-effects (RT_probe_ − RT_neutral_ and ER_probe_ − ER_neutral_). The analyses only revealed small and non-significant correlations for RTs with *r* = 0.08, *p* = 0.32 and ER with *r* = 0.01, *p* = 0.95. Figure 1 illustrates the variability in the data for both the RT CIT-effect as well as the BAC, but also highlights that this correlative approach might be inappropriate here due to the large number of participants with a BAC of zero.

To gain more information on our null result in the interaction effect of the ANOVAs, we performed two Bayesian independent samples *t* tests comparing the CIT-effects in ERs and RTs between the Alcohol and the Control group. The Bayes Factors (BFs) were calculated using the open source software JASP (https://jasp-stats.org/), using a JZS-prior (chouchy prior of 0.707) for the alternative hypothesis. The BFs reporting the evidence for the null-hypothesis BF_01_ were 5.99 and 3.66 for the CIT-effects in the ERs and RTs, respectively, suggesting that our data is 5.99 and 3.66 more likely to be observed under the null hypothesis than the alternative hyothesis.

To explore any further potential influences on the CIT-effect, correlations were computed between RT and ER CIT-effects and feelings of tension, boredom, fatigue, intoxication, the perceived difficulty of the test, and test scores in the BIS-11, the AUDIT, the DAST-10, the PPI-R and the number of lies in the last 24 h and the last week (with *p* being corrected for the number of tests: *p*/22). None of those were statistically significant.

## Discussion

The aim of the current experiment was to test whether acute alcohol intoxication, which was associated with increased CIT-effects in a field study by Suchotzki and Gamer^6^ would have similar effects in a controlled laboratory setup. For this purpose, participants in the current study did not choose whether they consumed alcohol or not by themselves, but were randomly assigned to an alcohol or a sober control condition. Interestingly, no effects of alcohol intoxication on CIT-effects were observed. As indicated by the Bayes Factor, this provides at least moderate evidence for the null hypothesis^17^. This indication, as well as our sample size (which was more than twice the size required to detect an effect as large as in Suchotzki and Gamer^6^ with a power of 0.95), indicates that the observed null-effect is most likely attributed to a genuine absence of such an effect, at least in conditions resembling the current study. Aside from the possibility that effects observed in the previous study with a considerably smaller *n* of 42 were due to chance, several other theoretically interesting explanations for the discrepancy between the current results and the results of Suchotzki and Gamer^6^ are possible.

First, as mentioned in the introduction, the randomized assignment of participants in the current study controls for potentially confounding effects of habitual alcohol use. However, the small and statistically non-significant correlations in the current study between participant’s habitual alcohol use and the CIT-effects speak against this notion to fully explain the diverging results (AUDIT*RT CIT-effect *r* = − 0.07; AUDIT*ER CIT-effect *r* = 0.00). Nevertheless, the combination of habitual and acute alcohol consumption at the time of testing may be necessary for generating impairments in executive functioning, thus lowering performance in the CIT. Aside from this, alcohol intake in a laboratory environment differs from realistic drinking environments in many other aspects, with regard to different environmental cues, social factors, and reinforcing effects of alcohol intake. Accordingly, as revealed by a meta-analysis of McKay and Schare^18^, both pharmacological as well as expectation effects of alcohol intake seem to be moderated by the experimental setting (in the meta-analysis experimental vs. natural vs. bar setting). Such moderation might also account for the discrepancy between our lab-based setup and the previous field study.

A second difference between both investigations concerns the average BAC levels and their distribution. In the field study of Suchotzki and Gamer^6^, the average BAC was 0.06% across all participants, whereas in the current study it was only 0.02% across all participants (and 0.04% in the alcohol group). Similarly reflecting the differing voluntary alcohol intake in the field study, the BAC variance there was larger (with *SD* = 0.05) than in the current lab study (with *SD* = 0.02). Of course, there are ethical and practical limitations to how much alcohol should be administered in laboratory studies, but although other studies have shown that executive functioning can already be impaired at moderate intoxication levels (~ 0.04%, for a review see^9^), the lower BAC average in the current study could also explain the absence of an effect. Third, motivational factors may also have counteracted effects of the alcohol intoxication^19,20^, as motivation has shown to improve participants’ performance in reaction time-based deception and concealed information paradigms^21^. Although ratings of boredom did not differ between alcohol and control group in the current study, lower ratings of fatigue in the alcohol group could possibly reflect an increased motivation and thus increased efforts of intoxicated participants to counteract alcohol effects.

Asking participants afterwards whether they believed to have been in the alcohol or the control group revealed that 94% of the alcohol group correctly believed to be in the alcohol group, yet also 44% of the control group incorrectly believed to be in the alcohol group. Thus, the “blinding” seemed to have worked only to a certain extent. The goal of such a blinding procedure would have been to disentangle pharmacological effects of alcohol from expectancy effects, yet this may have been unnecessary in our study for two reasons. First, real-life alcohol effects are certainly comprised of both—pharmacological and expectancy effects—and those real-life effects were of primary interest in this study. Second, as we did not observe any effects of alcohol intake on the CIT-effect in our study, disentangling the two (pharmacological and expectancy effects) seems unnecessary. However, this research question still seems interesting for follow-up studies on potential alcohol effects in the CIT.

In summary, even though theoretical accounts on the involvement of executive functions during the RT CIT^10^ and previous findings^6–8^ suggested larger RT CIT-effects during alcohol intoxication, this was not the case in the current study. Based on our well-powered sample and the careful experimental design in which participants were randomly assigned to either an alcohol or a sober control group, this finding is certainly a strong indicator for the absence of an effect under the current circumstances. Examining the differences between field and laboratory environments serves as guide for future research to identify factors moderating the potentially more complex relationship between alcohol intoxication and CIT-performance. As detailed above, candidates for such moderators are personality traits that are closely linked to voluntary alcohol consumption and habitual alcohol intake, as well as environmental aspects like the social context in which drinking takes place. In addition, even if the amount of alcohol given in laboratory contexts is (and should be) clearly restricted by ethical considerations, future research may find ways to study potential dose-dependent effects of alcohol on information concealment.

Finally, the question about potential underlying theoretical mechanisms deserves further attention. The fact that we did not employ a more specific measure of executive functioning is certainly a limitation of the current study. Previous research has shown impairments of executive functions already at the moderate BAC levels achieved in the current study^9^, yet it remains unclear whether similar effects were also evident here. Future research could even implement different measures for individual facets of executive functioning such as working memory, task switching, and the different aspects of response interference and inhibition, in order to additionally examine the unanswered question regarding the cognitive functions specifically involved in information concealment^10,22–27^.

To conclude, the fact that we did not observe an effect of moderate alcohol intoxication on the RT CIT-effect when employing a controlled randomization procedure in a lab-based test-environment should motivate further research to take a closer look at potential moderating factors. At the same time, our findings of robust RT CIT-effects across experimental and control groups substantiate the notion that the CIT can be applied across various conditions with moderate alcohol intoxication having neither beneficial nor deleterious effects on CIT validity. These findings thus undermine the occasionally heard argument that alcohol may serve as a countermeasure in applied CIT investigations^28^, at least when an RT-based CIT is used.

## Data Availability

Raw data, the preprocessing script and the aggregated data can be accessed on https://osf.io/g3t7q/.
